# Learning From Mistakes: The Role of Phages in Pandemics

**DOI:** 10.3389/fmicb.2021.653107

**Published:** 2021-03-17

**Authors:** Ahlam Alsaadi, Beatriz Beamud, Maheswaran Easwaran, Fatma Abdelrahman, Ayman El-Shibiny, Majed F. Alghoribi, Pilar Domingo-Calap

**Affiliations:** ^1^Department of Veterinary and Animal Sciences, University of Copenhagen, Frederiksberg, Denmark; ^2^Institute for Integrative Systems Biology, I2SysBio, Universitat de València-CSIC, Paterna, Spain; ^3^FISABIO-Salud Pública, Generalitat Valenciana, Valencia, Spain; ^4^Department of Biomedical Engineering, Sethu Institute of Technology, Rajapalayam, India; ^5^Center for Microbiology and Phage Therapy, Biomedical Sciences, Zewail City of Science and Technology, Giza, Egypt; ^6^Infectious Diseases Research Department, King Abdullah International Medical Research Center, Riyadh, Saudi Arabia; ^7^King Saud bin Abdulaziz University for Health Sciences, Riyadh, Saudi Arabia; ^8^Department of Genetics, Universitat de València, Paterna, Spain

**Keywords:** multidrug-resistant bacteria, phage therapy, public health, emergent pathogen, antibiotic resistance

## Abstract

The misuse of antibiotics is leading to the emergence of multidrug-resistant (MDR) bacteria, and in the absence of available treatments, this has become a major global threat. In the middle of the recent severe acute respiratory coronavirus 2 (SARS-CoV-2) pandemic, which has challenged the whole world, the emergence of MDR bacteria is increasing due to prophylactic administration of antibiotics to intensive care unit patients to prevent secondary bacterial infections. This is just an example underscoring the need to seek alternative treatments against MDR bacteria. To this end, phage therapy has been proposed as a promising tool. However, further research in the field is mandatory to assure safety protocols and to develop appropriate regulations for its use in clinics. This requires investing more in such non-conventional or alternative therapeutic approaches, to develop new treatment regimens capable of reducing the emergence of MDR and preventing future global public health concerns that could lead to incalculable human and economic losses.

## Introduction

The current global health emergency caused by severe acute respiratory coronavirus 2 (SARS-CoV-2), the causative agent of coronavirus disease (COVID-19), highlights the challenge to combat emergent pathogens with uncharacterized pathogenesis, limited treatments, and unavailable vaccines. The rapid and uncontrollable geographic dissemination of SARS-CoV-2, which has hit almost every country on earth, is still ongoing (Lai et al., 2020). As another example, recent Ebola virus outbreaks in different African countries, with low transmissibility but high mortality rates (around 50%), also put the already fragile health system in those countries under immense stress (Delamou et al., 2017). These two outbreaks reflect the need to invest more in science, and the importance of basic and translational research to improve public health and become ready for future pandemics. It is thus of particular interest to increase the scientific knowledge of better prevention, rapid detection, and effective treatment of emerging and/or re-emerging pathogens. Emerging zoonotic diseases are a growing public health concern and require close monitoring to understand the mode of transmission between people and animals. Thus, the “One Health” approach, with the aim of monitoring and integrating animal and human diseases through a systematic surveillance program (Mackenzie and Jeggo, 2019), will provide a better understanding to control zoonotic infections and enable rapid outbreak detection.

Bacterial infections, despite their longer replication times compared to viruses, are also responsible for high mortality and morbidity rates worldwide (O’Neill, 2014). The emergence of multidrug-resistant (MDR) bacteria is a significant concern nowadays (Lomazzi et al., 2019), and international institutions such as the WHO, the Centers for Disease Control and Prevention (CDC), or the European Commission, are calling for research into the development of alternative treatments (Binns, 2020). To date, infections from untreatable bacteria known as pan-resistant pathogens, resistant to all available classes of antibiotics, have been reported in many countries (David et al., 2019; Havenga et al., 2019). This has led to a high-priority challenge of understanding and countering progressive antibiotic resistance both in nosocomial and non-nosocomial settings. Thus, alternative therapies to treat and control MDR bacterial infections are highly desired in medicine today. One alternative that is gaining attention is bacteriophages (phages) due to their therapeutic potential administer alone (Martinecz and Wojewodzic, 2020), in combination with antibiotics (Manohar et al., 2020), or by interfering with eukaryotic viruses (Górski et al., 2020). It is time to learn from mistakes and invest in research against MDR bacteria. Prevention in health is not only about saving lives but also avoiding economic, social, or cultural burdens.

## The Emergence of MDR Bacteria and its Impact on Public Health

The discovery of antibiotics was a revolution in medicine. Since then, antibiotics have become crucial in the healthcare system, and have helped to improve the quality of life for humans and increased life expectancy. However, bacteria can be resistant to antibiotics through genomic alteration or acquiring mobile genetic elements harboring resistance and virulence-related genes (Oz et al., 2014; Baym et al., 2016; Reygaert, 2018; Wistrand-Yuen et al., 2018). The widespread misuse of antibiotics has fostered the selection of antibiotic-resistant bacteria and now infections due to antimicrobial-resistant strains are considered the second leading cause of death worldwide by killing around 7,000,000 people a year (O’Neill, 2014). According to the WHO, 10 million MDR deaths are expected in 2050, exceeding cancer, and it would cost the world up to 100 trillion USD (O’Neill, 2014; de Kraker et al., 2016). These expectations disregarded the current health crisis caused by COVID-19 that might exacerbate the worst scenario (Monnet and Harbarth, 2020). Bacterial co-secondary infections are common when a viral infection is established and they further increase morbidity and mortality (Morens et al., 2008; Smith and McCullers, 2014; Morris et al., 2017). Similar results were observed in 2009 during the H1N1 influenza virus outbreak, where bacterial infections contributed to 50% of the total deaths (Papanicolaou, 2013). The on-going pandemic is not an exception, and it has been shown that around 50% of patients who died from COVID-19 had secondary bacterial infections (Zhou et al., 2020). Also, it has been observed that critical patients had a higher percentage of bacterial coinfections (34.5%) than patients with severe or moderate disease (8.3 and 3.9%, respectively), increasing the mortality rate and duration of hospitalization (Feng et al., 2020). These infections include MDR bacteria (Lescure et al., 2020; Sharifipour et al., 2020), although their extent over the total cases remains unknown (Clancy et al., 2020). However, they are expected to be relevant given the high percentage of patients admitted to the intensive care units, where MDR infections are known to be common (Vincent et al., 2020). In fact, half of the hospital-acquired infections in COVID-19 patients occurred in critical units (Rawson et al., 2020). These are clear examples of the devastating effects of bacterial infections in the face of pandemics caused by viruses. In these cases, broad-spectrum antibiotics are misused to avoid undesirable secondary infections (Deng and Peng, 2020). For instance, in a random cohort of 1,705 patients of Michigan hospitals, antibiotics were prescribed to 57% of COVID-19 hospitalized patients, while only 3.5% had a confirmed bacterial co-infection (Vaughn et al., 2020). This was even worse in hospitals from The Netherlands, where bacterial infections were rare (1.2%), but more than 60% of patients received antibiotics (Karami et al., 2021). Thus, even if the frequency of bacterial infections seems to be low in the SARS-CoV-2 pandemics, this indiscriminate use of antibiotics fosters the spread of MDR bacteria (Rawson et al., 2020). Maybe this effect could be compensated due to travel restrictions and better sanitation practices in developed countries, but the increase of MDR bacteria in places with poor health systems seems unavoidable (Collignon and Beggs, 2020; Egyir et al., 2020; Monnet and Harbarth, 2020).

Lastly, it is also worth mentioning that the extensive use of antibiotics in farming is contributing to the spread of antibiotic resistance. Antibiotics are used in animals for growth promotion and for the prevention of diseases. This usage is responsible for 80% of total antibiotic consumption in the United States and is predicted to rise by 2030 (Van Boeckel et al., 2015). This has led to isolating MDR bacteria from food animals (Zhu et al., 2013) and livestock wastes (He et al., 2020), promoting their dissemination into environments where humans can be exposed, thus increasing antibiotic-resistance (Manaia, 2017). For example, several bacterial pathogens from the food animals can cause several health issues in humans (Strawn et al., 2013; Hellberg and Chu, 2016; Chlebicz and Śliżewska, 2018), being a major reservoir of antibiotic-resistance that can be transferred to humans directly by the food chain. Given the scarce development of new antibiotics due to resistance predictability, economic incentives, and regulatory requirements over the last years (Ventola, 2015), non-canonical alternatives might pave the road.

## The Need to Explore Alternative Treatments: Phage Therapy in the Spotlight

Phages, viruses that infect bacteria, are the most abundant biological entities on Earth and can be found everywhere (soil, oceans, human gut, sewage, and wastewater), being stable in many conditions (Domingo-Calap and Delgado-Martínez, 2018). They were postulated since their discovery as therapeutic tools against pathogenic bacteria (D’Herelle, 2007) since they can be isolated and used against bacterial pathogens (Moye et al., 2018; Jamal et al., 2019). Interestingly, phage abundance, versatility, ubiquity, and genetic diversity are beneficial for their use as therapeutic tools, making phage discovery a rapid, simple, and limitless process (Altamirano and Barr, 2019).

The highly specific interaction between phage and host, allow them to recognize and lyse specifically the targeted bacteria (Hyman and Abedon, 2010), being safe for humans and animals, and avoiding dysbiosis (Skurnik et al., 2007). Interestingly, although many phages can infect a narrow range of bacteria (closely related strains; Hyman and Abedon, 2010), some phages have a broad host range, meaning that they can infect multiple species of bacteria or multiple strains of the same bacterial species (Mirzaei and Nilsson, 2015).

However, the arms race between phage and bacteria takes place as an evolutionary trade-off. Bacteria have evolved resistance mechanisms to combat phage infection, whereas phages have developed a wide array of mechanisms to overcome bacterial defense systems (Oechslin, 2018). Phage-resistant mechanisms in bacteria include their ability to alter or mask the cell wall receptors or block the phage DNA entry. By the activation of the CRISPR-Cas system, the viral DNA in the cell is degraded upon phage infection. However, to encounter these mechanisms, phages can recognize new or the altered receptors and escape CRISPR-Cas resistance through anti-CRISPR viral encoded proteins (Pawluk et al., 2018).

Under this view, phages should be considered as promising adaptable tools in the fight against multidrug-resistant bacteria, especially nowadays, where the recent pandemics have increased the hospitalization pressure, and nosocomial infections are rising fast without effective treatments. Indeed, the potential use of phages and phage-derived enzymes in COVID-19 patients has recently been highlighted (Martinecz and Wojewodzic, 2020). We consider that phages could be an interesting alternative to combat bacterial pathogens, and we strongly encourage increased investment in the field. Further research in phage biology of novel discovered phages, phage-resistance emergence, phage stability, pharmacokinetics and pharmacodynamics, legislation, and regulation, should be addressed prior to its implementation as a routine therapy.

## Improving Phage Potential Through Genetic Engineering

Outbreaks of MDR bacteria challenge the efficacy of phage therapy applications due to bacteria’s ability to adapt and develop resistance to phages (Labrie et al., 2010). Whole-genome sequencing (WGS) of clinical bacterial genomes isolated from patients could reveal the presence of prophages in the chromosome, phage resistance encoding genes, or genetic modifications in the genome to encounter phage infections (Donkor, 2013). Thus, bacterial genomic features and screening for prophages will improve the phage therapy efficacy. As for phage or phage-derived product use, WGS has become mandatory for regulatory approval in both healthcare and food-industry (Hayes et al., 2017; Aziz et al., 2018).

Characterizing the phage genome is important for therapeutic applications. The phage genomes are hyper-mobile and it is essential to recognize their properties for a safe phage therapy selection (Loc-Carrillo and Abedon, 2011). Screening includes the phage life cycle-related genes such as integrase genes, and the lack of deleterious genes besides the presence/absence of phage genes that encode for virulence and transducible elements such as antibiotic resistance genes. Genetic transfer *via* transduction is a defined phage biology by-product by which the phage mediates the transfer of antibiotics resistance genes, virulence factors, and fitness-related genes as an evolutionary trait.

Essential phage properties can be evaluated experimentally as well as can be analyzed by computational methods (McNair et al., 2012). The advances in high-throughput genome sequencing, along with the development of CRISPR-Cas and other recombineering techniques, have opened a wide range of opportunities to improve phage intrinsic characteristics. Their smaller genome size and ease of propagation and manipulation make them ideal candidates subjected to genetic manipulation (Litman and Pardee, 1956; Freese, 1959). However, the impact of engineered phages on bacterial and phage community dynamics are not understood (Nair and Khairnar, 2019). Genetic modification of phages is mainly applied to address some of the drawbacks of their clinical application as narrow host range, lysogeny, or efficacy. Phage host range firstly relies on tail fiber and base plate proteins, which are responsible for recognizing cell surface receptors. Therefore, these proteins are ideal targets to alter a phage host-range. Thus, it is possible to expand the phage host range, select those less prone to bacterial resistance or extended tropism (Dunne et al., 2019). In clinical settings, the host range specificity supports the approach of administering phages as therapeutic agents. The approach of using a single phage to infect a bacterium requires precise matching between the phage and the bacterial host. However, phage cocktails can increase phage host range, and reduce the emergence of phage-resistance variants. Cocktails can be composed of phages infecting a single bacterial strain, multiple strains, or multiple species (Abedon, 2017). With consideration of phage pharmacokinetic and phage-host interaction, sequential administration of phages to patients at different times during the course of treatment has been proposed. This approach could reduce bacterial resistance rates caused by modifications in host cell receptors, which prevent phage infection (Nilsson, 2014; Mapes et al., 2016). Interestingly, with the advance of phage bioengineering techniques, it is possible to direct phages at specific bacteria. Several studies use customized phages to re-sensitize or remove antibiotic-resistance pathogens from the mixed bacterial populations (Dunne et al., 2019). These techniques support the phage host range modification to broaden the phage-host interaction and make it achievable.

Temperate or lysogenic phages are not desired for phage therapy as they get integrated into the host genome, thus limiting their efficacy and increasing the risk for horizontal gene transfer. As above, temperate phages could be switched to lytic phages by removing factors that allow for lysogeny such as integrases and repressors. This approach was used to treat a patient with a disseminated drug-resistant *Mycobacterium abscessus* and represented the first case of phage therapy using an engineered phage (Dedrick et al., 2019).

Lastly, one of the pitfalls of phage therapy so far has been stability and storage conditions. For that purpose, phages have also been manipulated to resist different pH conditions for example, displaying lipids on their surfaces (Nobrega et al., 2016), or increase thermal stability (Favor et al., 2020). Alternative approaches such as the engineering of phage proteins can be used instead since advances in phage protein engineering opens up new means to fight MDR bacteria more efficiently (Gerstmans et al., 2020). It is also worth mentioning phage display, a promising technique and powerful influential tool in molecular biology, based on the display of a unique peptide sequence or protein on the outer surface of the phages by genetic fusion to coat proteins of phage virion (Smith, 1985). Phage display has a role in molecular biology, with widespread applications in therapeutics, highlighting drug discovery as a major application (Lowman, 1997; Mimmi et al., 2019), drug delivery (Karimi et al., 2016), cancer imaging (Ghosh et al., 2012), vaccine development (Bao et al., 2019), and treatment against infectious diseases (Bazan et al., 2012; Bao et al., 2019).

## Current Status of Phage Therapy as a Biomedical Tool

Since the first encouraging reports of phage therapy after phage discovery, a lot of studies have shown the potential and efficacy of phages to treat bacterial infections. However, most of them have been done *in vitro* or were not properly designed/documented to draw definitive conclusions (Domingo-Calap et al., 2016). During the last years, a growing amount of *in vivo* studies, mainly in mouse models have been carried out (Melo et al., 2020). Most of our knowledge of phage therapy came from the Eliava Institute of Bacteriophage, in Georgia, and the Ludwik Hirszfeld Institute of Immunology and Experimental Therapy, in Poland, where phage therapy is routinely used as an antibacterial treatment. In contrast, in Western Europe and the United States, a regulatory framework should be established, since there are still limited studies under the guidelines of the Food and Drug Administration (FDA) or European Medicines Agency (EMA), most of them as clinical reports of compassionate use, due to the lack of clinical trials.

As of December 2020, there are 46 terminated or active published clinical trials that contain the term “phage”.^1^ Of these, 19 are strictly related to phage therapy, and only one in phase II (NCT03140085), comparing the efficacy of a phage cocktail against urinary tract infections (Ujmajuridze et al., 2018). Unfortunately, no significant efficacy rate of the phage treatment was reported. Interestingly, no side effects of phage therapy were observed and supporting the lack of side effects of phages. These results are in line with the PhagoBurn trial, the world’s first prospective multicentric, randomized, single-blind, and controlled clinical trial of phage therapy (Jault et al., 2019). PhagoBurn trial focused on patients with burn wounds due to bacterial infections, administering a cocktail of 12 phages topically. The phage treatment resulted in bacterial reduction with less serious events in comparison to the standard care group. Yet, the results were less promising than expected. The authors have reasoned these results to the manufacturing-related process and administration challenges that caused the delay and the reduction of phage cocktail titer that led to patients receiving doses lower than originally intended. Design of clinical trials for phages should consider the unique features of these entities as they are self-replicating viruses. This is even more challenging when considering phage cocktails, as the interactions between the different phages, including storage, are usually poorly understood. Therefore, although some phage-based products are available in some Eastern European countries, there are no phage products approved for human therapy in the United States or the rest of Europe to date (Fauconnier, 2019; Pelfrene et al., 2019).

Meanwhile, a few cases of compassionate phage therapy have been reported. Compassionate treatment refers to the use of medicines outside of a clinical trial for a patient with unavailable approved therapeutic options and is under the “Helsinki Declaration of Ethical Principles for Medical Research Involving Human Subjects.” During the last two decades, more than 25 reports of the compassionate use of phage therapy after antibiotic failure have been published (McCallin et al., 2019; Schmidt, 2019) and might help in designing new clinical trials. In Belgium, this has led to the approved use of phages as ingredients of magistral preparations, thus as a means of personalized treatment (Pirnay et al., 2018). The compassionate and personalized use of phages, although positive to extend our knowledge about the efficacy of phage therapy *in vivo*, highly relies on the regulatory framework of each country. That is why a call-up has been made around the regulatory hurdles of phage therapy in order to get appropriate therapeutic guidelines that make the adaptation and implementation of phages in clinical settings easier (Moelling et al., 2018). Recently, the FDA has approved phage therapy as a compassionate treatment for COVID-19 patients (Adaptive Phage Therapeutics, Inc., 2020), due to the high incidence of MDR secondary infections mentioned previously.

## Need for a Phage-Based Regulatory Framework and Future Perspectives

The current regulatory framework worldwide is not allowing the use of phage therapy in Western Europe and the United States (Parracho et al., 2012). Hence, the phage community calls for new and specific standards to implement phage therapy. The phage therapy-based regulatory framework should include providing well-characterized phages, including isolation and purification, defining host range, sequencing, and storage in phage banks. These will provide an available well-defined collection ready-to-use for clinical care. Importantly, patients should be informed of the phage therapy and given the option to decide to try it. It is also worth mentioning that it is recommended by the FDA to follow phage manufacturing under Good Manufacturing Practice (GMP) guidelines and infrastructure. However, the implementation of GMP is considered for a large phage production whereas phage-specific patients are produced for a limited number of phages, thus, a simpler GMP system is recommended.

Phage banks are already increasing, and some of them are located in Belgium, Republic of Georgia, Russia, Germany, Switzerland, Finland, and Canada (Moelling et al., 2018). In addition, Phage Directory, an online database of phage laboratories, phages, and bacterial hosts, in which phage researchers, regulators, and biotech companies are communicated, is an interesting example of a network to implement the use of phages in clinics. This huge phage database provides an opportunity to continue working on research to form therapeutic phages, even as a form of individualized medicine.

Political authorities, stakeholders, academics, and researchers around the world must be aware of the need for urgency to treat a high number of people suffering from MDR infections, which are predicted to be much higher as a primary and as a secondary infection during the current pandemics, being a major global health threat. This can be achieved by the establishment of a phage therapy-related regulation to allow for phage therapy research development and to increase incentives in order to increase basic research and translate it to proper clinical trials.

## Conclusion

The emerging and growing threat of MDR bacteria is of public concern to both health and economic communities around the world. In the search for novel treatments against pathogenic bacteria, phages have been shown to be promising therapeutic tools. However, the therapeutic use of phages in clinics is currently a major challenge. Awareness of the importance of phage therapy is increasingly appreciated by society, including academics, physicians, patients, and social media. This should therefore open the door to more investment in phage research and increase support and funding for phage biology and the development of phage-based treatments, including clinical trials. In addition, it is significant to rebuild the regulatory frameworks with respect to phage therapy and its potential applications. In conclusion, we consider that anticipating health is winning, and long-term initiatives to prevent future global health outbreaks should be a major concern nowadays.

## Data Availability Statement

The original contributions presented in the study are included in the article/supplementary material, further inquiries can be directed to the corresponding authors.

## Author Contributions

AA and PD-C drafted the main parts of the manuscript. AA, BB, ME, FA, and AE-S contributed to parts of the manuscript. AA, MA, and PD-C reviewed and edited the manuscript. All authors contributed to the article and approved the submitted version.

### Conflict of Interest

The authors declare that the research was conducted in the absence of any commercial or financial relationships that could be construed as a potential conflict of interest.

## Footnotes

^1^https://clinicaltrials.gov/
